# Medulloblastoma, macrocephaly, and a pathogenic germline *PTEN* variant: Cause or coincidence?

**DOI:** 10.1002/mgg3.1302

**Published:** 2020-05-17

**Authors:** Jussi‐Pekka Tolonen, Anne Hekkala, Outi Kuismin, Hannu Tuominen, Maria Suo‐Palosaari, Olli Tynninen, Riitta Niinimäki

**Affiliations:** ^1^ Department of Pediatrics MRC Oulu PEDEGO Research Unit University of Oulu and Oulu University Hospital Oulu Finland; ^2^ Department of Clinical Genetics MRC Oulu PEDEGO Research Unit University of Oulu and Oulu University Hospital Oulu Finland; ^3^ Department of Pathology Cancer and Translational Medicine Research Unit University of Oulu and Oulu University Hospital Oulu Finland; ^4^ Department of Diagnostic Radiology Oulu University Hospital and University of Oulu Oulu Finland; ^5^ Research Unit of Medical Imaging, Physics and Technology Faculty of Medicine University of Oulu Oulu Finland; ^6^ Medical Research Center University of Oulu Oulu Finland; ^7^ Department of Pathology HUSLAB University of Helsinki and Helsinki University Hospital Helsinki Finland

**Keywords:** carcinogenesis, disease susceptibility, magnetic resonance imaging, medulloblastoma, megalencephaly

## Abstract

**Background:**

Medulloblastomas (MBs) are a heterogeneous group of childhood brain tumors with four consensus subgroups, namely MB_SHH_, MB_WNT_, MB_Group 3_, and MB_Group 4_, representing the second most common type of pediatric brain cancer after high‐grade gliomas. They suffer from a high prevalence of genetic predisposition with up to 20% of MB_SHH_ caused by germline mutations in only six genes. However, the spectrum of germline mutations in MB_SHH_ remains incomplete.

**Methods:**

Comprehensive Next‐Generation Sequencing panels of both tumor and patient blood samples were performed as molecular genetic characterization. The panels cover genes that are known to predispose to cancer.

**Results:**

Here, we report on a patient with a pathogenic germline *PTEN* variant resulting in an early stop codon p.(Glu7Argfs*4) (ClinVar ID: 480383). The patient developed macrocephaly and MB_SHH_, but reached remission with current treatment protocols.

**Conclusions:**

We propose that pathogenic *PTEN* variants may predispose to medulloblastoma, and show that remission was reached with current treatment protocols. The *PTEN* gene should be included in the genetic testing provided to patients who develop medulloblastoma at an early age. We recommend brain magnetic resonance imaging upon an unexpected acceleration of growth of head circumference for pediatric patients harboring pathogenic germline *PTEN* variants.

## Graphical abstract

Pathogenic germline variants of the *PTEN* gene may predispose to medulloblastoma at an early age. We show that a patient carrying a pathogenic *PTEN* variant reached remission with current treatment protocols. We propose follow‐up guidelines to patients shown to carry pathogenic *PTEN* variants.

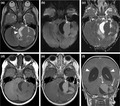

## 1 INTRODUCTION

Medulloblastoma (MB), a heterogeneous solid tumor of the posterior fossa, is the second most common malignant pediatric brain tumor after high‐grade gliomas (Millard & De Braganca, 2016; Ostrom et al., 2015). Typically, medulloblastomas (MBs) are aggressive and metastasize within the central nervous system and, less frequently, outside the neuraxis (Millard & De Braganca, 2016). The four consensus subgroups, MB_SHH_, MB_WNT_, MB_Group3_, and MB_Group 4_, exhibit distinctive transcriptional and epigenetic signatures that define clinically relevant patient subsets (Vladiou et al., 2019). However, an incomplete understanding of the underlying molecular etiology in each subgroup has impeded the development of specific treatment modalities (Vladiou et al., 2019).

MBs account for roughly 10% of central nervous system malignancies in children between 0 and 14 years of age with a worldwide age‐adjusted incidence of 2.0–5.8 cases per 1,000,000 annually (Millard & De Braganca, 2016; Waszak et al., 2018). The incidence peaks in two age groups, namely in children between 3 to 4 years and 8 to 9 years of age (Millard & De Braganca, 2016; Ostrom et al., 2015). Extensive single‐cell transcriptomics have revealed that MBs originate from irregularities in early brain development (Vladiou et al., 2019), which may explain why at least 70% of MBs occur in the pediatric population (Millard & De Braganca, 2016).

A recent study demonstrated that the MB_SHH_ subgroup suffers from the highest prevalence of genetic predisposition (approximately 20%) with recognized germline driver mutations in *APC*, *BRCA2*, *PALB2*, *PTCH1*, *SUFU* (OMIM *607035), and *TP53* (Waszak et al., 2018). However, the spectrum of germline mutations in MB remains incomplete. Here, we present the first known case report of cerebellar medulloblastoma with an inactivating germline variant in the tumor suppressor *PTEN* (OMIM *601728). We suggest that pathogenic germline *PTEN* variants may predispose patients to the early development of medulloblastoma and show that current treatment protocols may prove effective despite the *PTEN* status. We recommend brain magnetic resonance imaging (MRI) upon an unexpected acceleration of growth of head circumference for pediatric patients known to harbor such variants of the *PTEN* gene.

## 2 RESULTS

### 2.1 Ethics statement

The patient and her legal guardians conferred an informed consent for the study. The study complies with the Helsinki Declaration of 1975.

### 2.2 Methods

Histological characterization of the tumor was performed at the Department of Pathology (Oulu University Hospital, Oulu, Finland). Molecular genetics studies were performed using a Next‐Generation Sequencing (NGS) panel at the Laboratory of Genetics, HUSLAB (Helsinki University Hospital, Helsinki, Finland). A comprehensive hereditary cancer NGS panel of blood sample DNA was performed at the Blueprint Genetics laboratory (Helsinki, Finland). The panel includes 116 genes known to confer a hereditary predisposition to cancer.

### 2.3 Case report

The index patient was referred to the Oulu University Hospital at 14 months of age due to a 2‐week history of torticollis and temper tantrums. She is the first child of her parents, and the pregnancy and delivery were uneventful. Her birth weight, height and head circumference (+1.7 *SD*) were normal at birth. However, at the age of 2.5 weeks she presented with macrocephaly (+4.0 *SD*). The subsequent brain ultrasound and MRI were normal. Since the age of 5 months, her head circumference continued to grow on a +5 to +5.2 *SD* curve. Brain MRI was repeated at the age of 6 months and brain ultrasound at the age of 7 and 9 months. All the findings were normal. Otherwise, she developed normally and achieved walking at 12 months of age.

**Figure 1:**
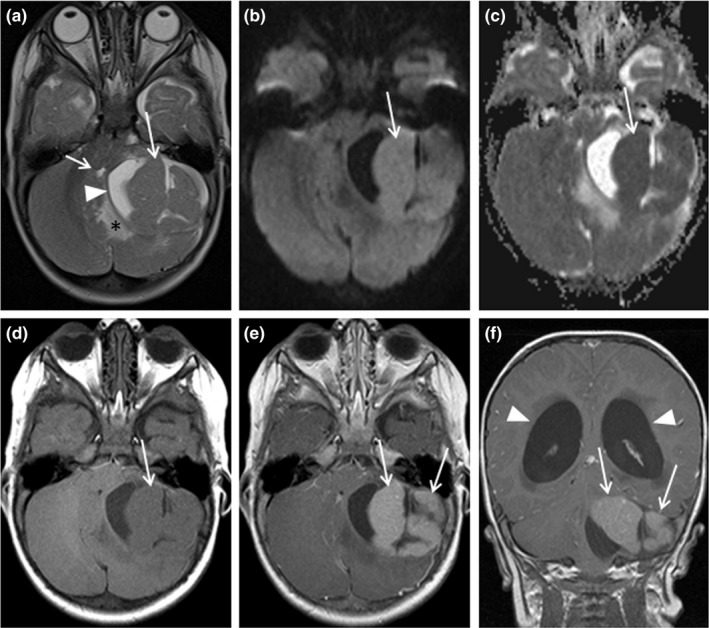
Brain MRI showing a tumor of the posterior fossa of a 14‐month‐old girl. T2‐weighted axial image (a) demonstrates a large cerebellar intraparenchymal hemispheric tumor with T2‐hyperintense cystic (arrowhead) and T2‐hypointense nodular solid (long arrow) components causing compression of the 4th ventricle (short arrow) and surrounding edema of the cerebellum (asterisk). Restricted diffusion of the solid components (b and c, arrows) suggesting highly cellular tumor is demonstrated on diffusion‐weighted imaging (b) with apparent diffusion coefficient (ADC) map (c). The solid component is slightly hypointense compared to cerebellum on native T1‐weighted image (d, arrow) but it enhances intensely with gadolinium contrast media showing nodular arrangement on contrast‐enhanced T1‐weighted axial (e) and coronal (f) images (arrows), which is indicative of desmoplastic nodular medulloblastoma. Lateral ventricles are dilated due to the compression of the tumor on the 4th ventricle (f, arrowheads). MRI, magnetic resonance imaging

By admission to the hospital, the child had regressed to cruising. Macrocephaly had progressed (+6.3 *SD*) and MRI using a 1.5 Tesla scanner revealed a large cerebellar tumor (4.0 × 5.5 × 6.5 cm in size) with obstructive hydrocephalus (Figure 1). The tumor was suspected to represent desmoplastic‐nodular medulloblastoma by nodular MRI features. Subsequently, the patient underwent immediate ventriculostomy and resection of the tumor mass. Complete resection was confirmed macroscopically and by postoperative MRI, and the child recovered as expected.

Post‐surgical chemotherapy was performed according to the SKK protocol in the HIT‐MED Guidance of the University of Hamburg‐Eppendorf for M0 tumors (with no postoperative residual tumor or metastases), between September 2017 and August 2018. Chemotherapy included Cyclophosphamide (1,200 mg/m^2^), Vincristine (16.5 mg/m^2^), Carboplatin (3,000 mg/m^2^), Etoposide (2,250 mg/m^2^), and Methotrexate (30,000 mg/m^2^). The child is currently in remission at 3.5 years of age.

By NGS analysis, the tumor was shown to be wildtype for the *PTCH1*, *SMO* and *TP53* genes, and *MYC* and *MYCN* amplifications were excluded. However, a *SUFU* (c.(412delinsCC), p.(Ala138Profs*32), NM_016169.3) mutation was discovered in an extended NGS panel confirming the MB_SHH_ subtype (i.e., medulloblastoma, SHH‐activated and *TP53* wildtype). Additional mutations were found in the *KDR* (OMIM *191306, c.(787C>A), p.(Pro263Thr), NM_002253.2) and *PTEN* (c.(18dup), p.(Glu7Argfs*4), NM_000314.4) genes. The *KDR* variant is classified as pathogenic (COSM353283) but is unlikely to be significant here, since the developing medulloblastoma benefits from a functioning *KDR* gene (Slongo et al., 2007).

Finally, genetic counselling was provided as per the HIT‐MED Guidance for MB_SHH_. A DNA sample extracted from the patient's blood was sent for sequencing. No known disease‐causing germline variants associated with medulloblastoma were identified, including the *APC*, *BRCA2*, *PALB2*, *PTCH1*, *SUFU*, and *TP53* genes (Waszak et al., 2018). However, the panel revealed a de novo heterozygous *PTEN* frameshift variant (c.(18dup), p.(Glu7Argfs*4)) leading to a premature stop codon and representing the same mutation observed in the tumor tissue. The *PTEN* variant has been classified as pathogenic (ClinVar ID: 480383). Another patient with a germline *PTEN* variant and medulloblastoma at 1.0 years of age has previously been identified by Waszak and colleagues (Table 1) (Waszak et al., 2018). Gröbner *et al*. have reported two patients with germline *PTEN* variants and medulloblastoma at 19 and 23 years of age (Gröbner et al., 2018).

**Table 1:** Known germline *PTEN* variants associated with MB at infancy

Age	Sex	Medulloblastoma subtype	Variant (cDNA)	Variant (protein)	Pathogenicity	ClinVar
1.0^a^	F	SHH	c.(856delA)	p.(Thr286ProfsTer5)	NA	NA
1.2	F	SHH	c.(18dup)	p.(Glu7Argfs*4)	Pathogenic	480383

Abbreviations: NA, not available; SHH, MB_SHH_.

^a^ Waszak et al. (2018).

## 3 DISCUSSION

We present the first known case report of a toddler carrying a pathogenic germline *PTEN* variant with macrocephaly and MB_SHH_. While macrocephaly (+3.3 to 5.5 *SD* in males and +2.9 to 4.1 *SD* in females) is a known diagnostic feature of germline *PTEN* variants (Plamper, Gohlke, Schreiner, & Woelfe, 2019; Tan, Mankad, Goncalves, Talenti, & Alexia, 2018), no imaging recommendations exist for follow‐up in macrocephaly (*i.e*., head circumference higher than 2 *SD* above the mean), particularly for patients with a positive family history of macrocephaly and no risk factors or neurological symptoms (Sampson, Berg, Huber, & Olgun, 2019; Tan et al., 2018). However, we suggest that *PTEN* variants may cause a predisposition to early medulloblastoma with implications for the timing of imaging studies.

The *PTEN* gene encodes a tumor suppressor protein with lipid and tyrosine phosphatase activities, and other non‐enzymatic roles (Lee, Chen, & Pandolfi pp., 2018; Pilarski, 2019). Its main mode of action is to oppose the PI3K‐AKT‐mTOR pathway to regulate cell proliferation and metabolism as a potent suppressor of tumorigenesis (Sampson et al., 2019). Interestingly, tumorigenesis is promoted in a continuum of loss in PTEN activity (i.e., from subtle changes in expression and interaction with other proteins, to inactivating mutations), which cancer cells achieve through multiple mechanisms (Lee et al., 2018). Thus, a heterozygous inactivation of the *PTEN* gene is sufficient to predispose to cancer (Lee et al., 2018; Liu, Wang, Wang, & Chan, 2019).

Consequently, point mutations in *PTEN* are observed in up to 37% of malignant tumors (Liu et al., 2019). The *PTEN* gene is associated with the PTEN hamartoma tumor syndrome including the overlapping Cowden (CS) and Bannayan‐Riley‐Ruvalcaba syndromes, the adult‐onset Lhermitte‐Duclos disease, and autism spectrum disorders with macrocephaly (Pilarski, 2019; Tan et al., 2012). Tan et al. (2012) have shown that patients with CS have an increased lifetime risk (6%–85.2%) for cancers of the breast, thyroid, endometrium, colorectum, and kidney, as well as melanoma, and a robust, evidence‐based follow‐up strategy of patients with germline *PTEN* mutations has been proposed. The current follow‐up protocols begin in adulthood or 5–10 years prior to the earliest incidence of a certain cancer type in the family (Eng, 2001). Notably, no cases of medulloblastoma were observed in the cohort of 368 patients harboring germline *PTEN* variants followed up by Tan and colleagues (Tan et al., 2012).

In contrast, recent studies implementing extensive NGS analyses have revealed that mutations in *PTEN* are also associated with adult and pediatric brain cancer (Gröbner et al., 2018; Robinson et al., 2012; Sturm et al., 2014; Waszak et al., 2018). A study by Gröbner et al. (2018) reported that 5% of a total of 42 cases of MB_SHH_ in their cohort were associated with germline *PTEN* variants, suggesting, in addition, that pediatric tumors demonstrate a rate of new mutations that is 14 times lower than that observed in adult cancer, suffering, instead, from a higher incidence of germline mutations (7.6%). Furthermore, their data suggest that 57% of pediatric cancer harbor a single driver mutation and that 50% of these mutations are druggable. Similarly, Waszak and colleagues established that germline mutations explain 5%–6% of medulloblastoma, most significantly in the *APC*, *BRCA2*, *PALB2*, *PTCH1*, *SUFU* and *TP53* genes, with *SUFU* and *PTCH1* mutations demonstrating a high prevalence in infant MB_SHH_ (Waszak et al., 2018). Their data include one pathogenic germline *PTEN* variant associated with MB_SHH_ (out of approximately 41 cases of MB_SHH_ with genetic predisposition) and show that familial history in MB_SHH_ with a germline mutation is uncommon. These data suggest that damaging germline variants in *PTEN*, although rare, do occur in medulloblastoma.

Because the cases of medulloblastoma that are linked with germline *PTEN* variants are so rare—and may thus represent coincidental association—additional evidence is required to elucidate the role of *PTEN* in medulloblastoma predisposition. Mechanistically, PTEN regulates a perivascular progenitor cell niche that may act as a platform for tumorigenesis (Zhu et al., 2016). The SHH and PI3K pathways converge to promote the proliferation of granule cell progenitors in the external granule layer of the cerebellum. The *Pten* ^−/−^ mouse model produced by Zhu and colleagues demonstrated irregular proliferation of the progenitors, which progressed to fully penetrant MBB_SHH_ with a co‐deletion of the *Tp53* gene. It is possible that *PTEN* variants predispose to a rapidly developing medulloblastoma but require a mutation in one or several “second genes” for tumorigenesis (Gröbner et al., 2018; Zhu et al., 2016). This might explain the infrequency of medulloblastoma in patients with germline *PTEN* variants. Accordingly, the extended NGS panel of our patient's tumor DNA revealed a point mutation in *SUFU* which has been implicated in the development of MB_SHH_ (Brugières et al., 2012; Waszak et al., 2018).

In conclusion, we report on a patient with macrocephaly and MB_SHH_ that developed before the first typical peak in medulloblastoma incidence at 3 to 4 years of age. Extensive NGS analyses revealed a pathogenic germline variant in *PTEN* and a second variant of the *SUFU* gene in the tumor DNA. Notably, remission was reached with current medulloblastoma treatment protocols despite the *PTEN* status. As certain countries are beginning to provide whole‐genome sequencing for all newly diagnosed pediatric cancer patients (Byrjalsen et al., 2018), coincidental germline variants may confound the search for true genetic predisposition in pediatric cancer. We therefore suggest that the genetic testing of young patients with medulloblastoma should include the *PTEN* gene in addition to the *APC*, *BRCA2*, *PALB2*, *PTCH1*, *SUFU* and *TP53* genes with well‐defined associations to medulloblastoma to characterize the role of *PTEN* in the development of medulloblastoma. Genetic counseling should be provided to families where germline mutations have been revealed.

## CONFLICT OF INTEREST

No potential conflicts of interest relevant to this article were reported.

## AUTHOR CONTRIBUTIONS

AH and RN performed clinical care of the patient. All authors contributed to the design of the study, including radiological, histological and genetic analyses. JPT, AH and MSP wrote the first draft of the manuscript. All authors contributed considerable input on the preparation and writing of the manuscript.

## Data Availability

The data that support the findings of this study are available on reasonable request from the corresponding author due to privacy restrictions.
